# Ophthalmic Vein Thrombosis Associated with Factor V Leiden and MTHFR Mutations

**DOI:** 10.3390/diagnostics13061052

**Published:** 2023-03-09

**Authors:** Cosmin Adrian Teodoru, Mihnea Munteanu, Nadina Mercea, Alina Moatar, Horia Stanca, Florina Georgeta Popescu, Horațiu Dura, Adrian Hașegan, Doina Ileana Giurgiu, Maria-Emilia Cerghedean-Florea

**Affiliations:** 1 Faculty of Medicine, “Lucian Blaga” University Sibiu, 550169 Sibiu, Romania; 2 Department of Ophthalmology, “Victor Babes” University of Medicine and Pharmacy, 300041 Timisoara, Romania; 3 Ophthalmology Clinic, Municipal Clinical Emergency Hospital, 300254 Timisoara, Romania; 4 Department of Ophthalmology, “Carol Davila” University of Medicine and Pharmacy, 050474 Bucharest, Romania; 5 Department of Occupational Medicine, “Victor Babes” University of Medicine and Pharmacy, 300041 Timisoara, Romania

**Keywords:** ophthalmic vein thrombosis, Factor V Leiden, MTHFR mutations, hyperhomocysteinemia

## Abstract

Superior ophthalmic vein thrombosis (SOVT) is a rare clinical entity that may be associated with hypercoagulability status. We present a case of a 77-year-old woman who presented to the emergency department complaining of eye ptosis, chemosis and conjunctival congestion in the right eye (RE). The ophthalmological examination revealed best-corrected visual acuity (BCVA) was 0.5 for the right eye (RE) 0.5 and 0.06 for the left eye (LE). Intraocular pressure (IOP) was 25 mmHg in RE and 14 mmHg in LE. Non-contrast computed tomography (CT) of the brain and orbits revealed a hyperreflectivity at the level of the right ophthalmic vein and inferior rectus muscle hypertrophy. An extensive hypercoagulable panel was completed and we found a positive result for Factor V Leiden (heterozygous mutation) and methyl-enetetrahydrofolate reductase (MTHFR-C677T homozygous mutations). Systemic steroidal anti-inflammatory and anticoagulant treatments were started immediately. Gradual resolution of symptoms was noted during the hospitalization, and BCVA in her RE was established at 0.7 at the 10-week follow-up.

Ophthalmic vein thrombosis is a rare clinical condition (with an incidence of 3–4 cases/million/year) but with particularly important effects on patients’ lives; therefore, early diagnosis and treatment are critical in these cases [1,2]. Clinical, ophthalmic vein thrombosis manifests with acute orbital signs such as unilateral ptosis, chemosis, ophthalmoplegia and decreased visual acuity [1].

Etiologically, it can be associated with a variety of septic pathologies such as orbital cellulitis, septic cavernous sinus thrombosis, or aseptic pathologies such as facial trauma or inflammation, carotid–cavernous fistulas, orbital neoplasms, autoimmune diseases or hypercoagulability status [2,3,4,5].

Superior ophthalmic vein thrombosis is a rare pathology in current practice with a multifactorial etiology [2]. Clinically, symptoms are often acute, with eye pain, chemosis, ptosis, conjunctival congestion and decreased visual acuity [6]. In these cases, risk factors can be local or systemic but usually include at least one component of the Virchow triad (hypercoagulability, hemodynamic changes (stasis, turbulence), endothelial injury/dysfunction) [7]. The diagnosis is usually based on CT or MRI results and according to the patient’s symptoms. In our case, CT scan revealed a hyperreflectivity at the level of the right ophthalmic vein and inferior rectus muscle hypertrophy (Figure 1).

Regarding the systemic factors involved, ophthalmic vein thrombosis has been associated with a variety of conditions such as hypercoagulability (thrombocytosis, antiphospholipid syndrome, hereditary hemorrhagic telangiectasia, use of contraceptive pills or pregnancy) [8]. Some studies in the literature describe the association of SOVT with other systemic inflammatory diseases (Graves’ disease, systemic lupus erythematosus, sarcoidosis, amyloidosis or vasculitis) [2,4,9,10,11,12,13].

An increased homocysteine level may be associated with this condition. Methylenetetrahydrofolate reductase (MTHFR) normally catalyzes the conversion of 5,10-methyltetrahydrofolate to 5-methyltetrahydrofolate. Even with a normal level of folic acid in the body, MTHFR activity slows down when the MTHFR gene C677T polymorphism is present. As a result, hyperhomocysteinemia can produce severe damage to the vascular endothelial cells [14]. Some studies claim that hyperhomocysteinemia is independently associated with an increased risk of thrombosis [15]. 

Factor V Leiden mutation (FVL) is one of the most common genetic risk factors for venous thromboembolic disease. Factor V mutations are also known to potentiate the effect of MTHFR on deep vein thrombosis [16]. This mutation is a point mutation in the factor V gene in which glutamine is substituted for arginine at position 506. As a result, the risk of thrombosis rises due to the activated protein C resistance (APC-R). Functional resistance to APC-R assays and genetic testing using DNA-based techniques can both be used to identify the FVL mutation and differentiate between heterozygotes and homozygotes. In the Caucasian community, the FVL mutation was discovered to be the most prevalent inherited thrombophilic condition, accounting for up to 37% of venous thrombosis cases [17]. In our case, ophthalmic vein thrombosis may be a consequence of the synergism between the two hematological abnormalities.

There is a significant risk of developing other ocular complications due to the hematological mutation such as retinal vein occlusion (RVO) or retinal artery occlusion (RAO). It has been proven that this mutation is related to venous thromboses. Systemic inflammation leads to RVO through the induction of systemic hypercoagulability. Additionally, certain studies have demonstrated that hyperhomocysteinemia, particularly in patients with the MTHFR C677T gene variant, is a significant risk factor for RVO [14]. Regarding arterial occlusions, the connection between these events and the mutations of Factor V Leiden and MTHFR are still controversial. However, the presence of coagulation disorders should be suspected especially in young people [18] and cases where their simultaneous presence was proved were documented in the specialized literature [19,20].

The onset and the symptoms in the presented case were typical for this pathology with ptosis, chemosis and conjunctival congestion. In rare cases, thrombosis of the superior ophthalmic vein can progress to thrombosis of the cavernous sinus [5]. Most of the patients present with unilateral ocular complaints, although bilateral ocular involvement has been reported as well [21]. 

The treatment of thrombosis of the superior ophthalmic vein depends on the etiology. Additionally, it depends on the severity of the signs and symptoms and associated systemic diseases. It is important to rule out an infectious cause (that would require antibiotic treatment). In aseptic cases, anticoagulant treatment can be initiated, but only after an adequate assessment of the associated bleeding risks [17]. Additionally, treatment with corticosteroids can be useful in reducing orbital inflammation and congestion [22]. In the present case, the initiation of anticoagulant treatment together with systemic corticosteroid treatment significantly improved the patient’s condition, with a favorable evolution and remission of symptoms. Due to increased intraocular pressure, the patient also received ocular hypotensive treatment during hospitalization. The optical coherence tomography (OCT) of the RE shows normal RNFL thickness. Also, fundus examination was normal in RE (Figure 2). Many orbital diseases, particularly those involving proptosis, have been associated with increased intraocular pressure. The pathophysiological mechanism is complex. Increased orbital pressure can influence the intraocular pressure both directly by increasing hydrostatic pressure around the eyeball or indirectly by compressing the episcleral pressure and orbital veins, thus increasing venous pressure [23].

Thrombosis of the superior ophthalmic vein is a rare clinical entity but with an increased risk of morbidity if it is not discovered and treated in time. The etiology is vast, and differential diagnosis can be difficult; therefore, imaging examinations are essential for diagnosis. Treatment depends on the etiology and generally includes antibiotics, anti-inflammatories and anticoagulants. In our case, the evolution was favorable under treatment with restoration of visual acuity. An improvement was noticed immediately after the systemic steroid anti-inflammatory and anticoagulant therapy. This demonstrated the accuracy of the diagnosis. 

## Figures and Tables

**Figure 1:**
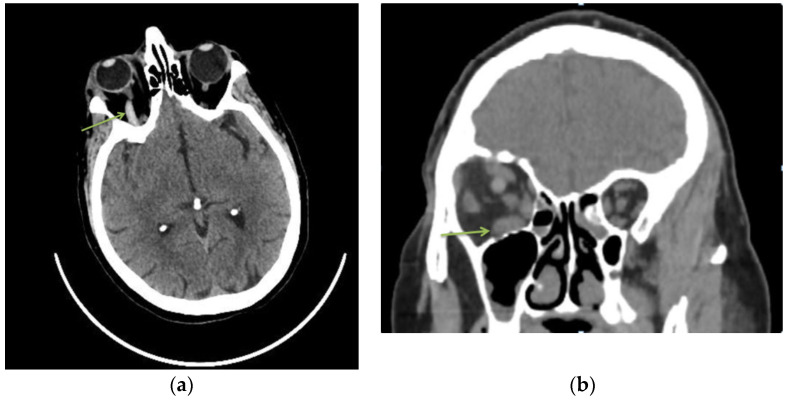
A 77-year-old woman, with hypertensive pathology, presented to the emergency department complaining of eye ptosis, chemosis and conjunctival congestion. The patient was known to have high myopia (both eyes) and amblyopia (LE). The ophthalmological examination revealed best-corrected visual acuity (BCVA) on the right eye 0.5 and 0.06 for left eye. Intraocular pressure (IOP) was 25 mmHg in RE and 14 mmHg in LE. The anterior segment of the left eye was normal. In the RE we noticed diffuse conjunctival congestion, chemosis and eyelid ptosis. Ocular movements were normal in both eyes. Pupillary light reflex was bilaterally intact. Hertel’s exophthalmometry revealed a proptosis of 20 mm in the right eye vs. 17 mm in the left eye. Non-contrast computed tomography (CT) of the brain and orbits revealed a hyperreflectivity at the level of the right ophthalmic vein (**a**) and inferior rectus muscle hypertrophy (**b**). Routine biochemical examination showed an increase of inflammatory markers (C-reactive protein, fibrinogen) and a high D-dimers value. Blood culture and a bacteriological conjunctival exam were negative. Additionally, other secondary causes were excluded (tumors, autoimmune diseases, infections). (**a**) Axial sections of non-contrast head CT scan—hyperreflectivity of the right ophthalmic vein. (**b**) Non-contrast head CT coronal scan—inferior rectus muscle hypertrophy.

**Figure 2:**
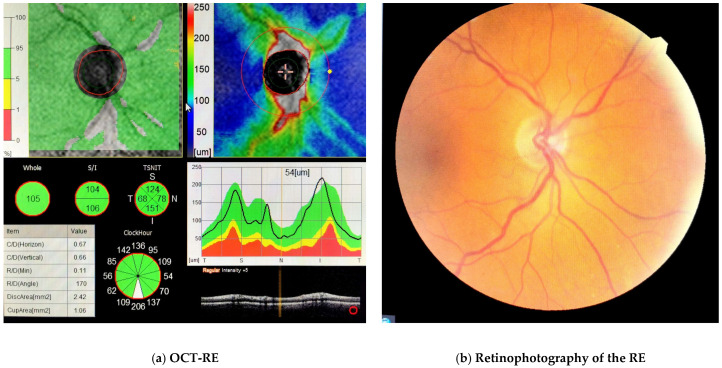
Optical coherence tomography (OCT) of the RE (**a**) showed no optic nerve damage and normal RNFL thickness. Additionally, fundus examination was normal in RE (**b**). The LE showed peripapillary atrophy of the optic nerve and a pigmented infero-nasal chorioretinal area. An extensive hypercoagulable panel was completed and we found a positive result for Factor V Leiden (heterozygous mutation) and MTHFR (C677T homozygous mutations). Additionally, a slight increase in the level of homocysteine was noted. Rheumatoid factor, antiphospholipid antibodies and antinuclear antibody were negative. Systemic steroid anti-inflammatory (dexamethasone 4 mg/mL twice a day) and anticoagulant (Enoxaparin 60 mg/0.6 mL twice a day) treatments were initiated along with antihypertensives (Perindopril 5 mg/once a day) and neuroprotector treatment for 10 days. She also received topical antibiotics and ocular hypotensive treatment (fixed combination of 2% dorzolamide/0.5% timolol (Cosopt)). During the treatment, INR, blood table and vital signs were monitored. Gradual resolution of symptoms was noted during the hospitalization, and the vision in her RE was preserved at 0.7 at the 10-week follow-up.

## Data Availability

The data presented in this study are available on request from the corresponding author.
